# The First Case(s) of Botulism in Vienna in 21 Years: A Case Report

**DOI:** 10.1155/2012/438989

**Published:** 2012-06-21

**Authors:** Matthias Gerhard Vossen, Klaus-Bernhard Gattringer, Judith Wenisch, Neda Khalifeh, Maria Koreny, Verena Spertini, Franz Allerberger, Wolfgang Graninger, Christian Kornschober, Heimo Lagler, Andreas Reitner, Thomas Sycha, Florian Thalhammer

**Affiliations:** ^1^Division of Infectious Diseases and Tropical Medicine, Department of Internal Medicine I, Medical University of Vienna, 1090 Vienna, Austria; ^2^Division of Nephrology and Dialysis, Department of Internal Medicine III, Medical University of Vienna, 1090 Vienna, Austria; ^3^Department of Emergency Medicine, Medical University of Vienna, 1090 Vienna, Austria; ^4^Clinical Institute of Hospital Hygiene, Medical University of Vienna, 1090 Vienna, Austria; ^5^Austrian Agency for Health and Food Safety, Institute for Medical Microbiology and Hygiene, 1220 Vienna, Austria; ^6^Austrian Agency for Health and Food Safety, Institute for Medical Microbiology and Hygiene, 8010 Graz, Austria; ^7^Department of Ophtalmology and Optometry, Medical University of Vienna, 1090 Vienna, Austria; ^8^Department of Neurology, Medical Universiy of Vienna, 1090 Vienna, Austria

## Abstract

We describe two linked cases of botulinum toxin intoxication to provide the clinician with a better idea about how botulism cases may present since early diagnosis and treatment are crucial in botulism. Botulinum toxin is the strongest neurotoxin known. *Methods:* We review the available literature, the compiled clinical data, and observations. *Results:* After a slow onset of clinical signs a married couple living in Vienna presented with dysphagia, difficulties in accommodation, inability to sweat, urinary and stool retention, dizziness, and nausea. They suffered intoxication with botulinum toxin type B. Botulism is a rarely occurring disease in Austria. In the last 21 years there were only twelve reported cases. *Conclusion:* Both patients went to a general practitioner as well as several specialists before they were sent to and correctly diagnosed at our outpatient department. To avoid long delays between intoxication and diagnosis we think it is crucial to advert to the complex symptoms a nonsevere intoxication with botulinum toxin can produce, especially since intoxications have become rare occurrences in the industrialized societies due to the high quality of industrial food production.

## 1. Introduction

Cases of botulism usually present with a descending flaccid paralysis, beginning in the bulbar muscles and involving at least one cranial nerve. The patients may complain about difficulty swallowing, a dry mouth, double vision, dysarthria, constipation and general fatigue. The onset of the symptoms usually takes place after two hours up to eight days after toxin ingestion [1]. Due to the rather unspecific presentation of botulism it is very important that physicians ask their patients if other people in their vicinity may show similar symptoms. 

Botulinum toxin is the strongest neurotoxin known. It can be divided into seven subcategories, labeled A to G, all of which are produced by bacteria of the *Clostridium botulinum *species. Botulinum toxin is an enzymatic inhibitor of acetylcholine release in synapses. A recovery is only possible if the motoneurons grow new axon twigs that reinnervate the muscle [1]. *Clostridium botulinum* itself is a spore-forming anaerobic bacterium usually found in soil. Classic cases of botulism developed from the ingestion of air-tight packed food in which *C. botulinum* was able to grow or from infected wounds. Although botulism was almost always fatal before the development of intensive care medicine, nowadays even intoxication with high doses of botulinum toxinum will not result in death if the patients are diagnosed and treated properly [2].

Botulism is a rarely occurring disease in Austria. In the last 21 years there were only twelve reported cases, none of which took place in Vienna (Table 1) [3–5]. 

Two patients, a married couple, both born in Serbia, but living in Vienna presented at our hospital. Both complained of a dry mouth and swallowing difficulties, newly developed blurred vision, inability to sweat, urinary and stool retention, dizziness, and nausea. The severity of the symptoms differed among both patients and was less pronounced in the wife. The husband additionally developed pharyngitis during the further course of the illness. 

## 2. Materials and Methods

We evaluated two linked cases of foodborne intoxication with botulinum toxin. Our case report was written by the attending physician after close review of the available literature and reexamination of the compiled clinical data and observations. 

## 3. Results and Discussion

The patients reported the onset of initial symptoms on two separate days, day zero and two, respectively. Both exhibited nausea as the first sign of intoxication, followed by diarrhea and vomiting in the husband's case. The patients reported an onset of blurred vision and increasing difficulties in swallowing on the 3rd day, after the onset of a prodromal nausea. Due to these symptoms the patients contacted a general practitioner, who referred them to an ophthalmologist, who prescribed glasses, and an otorhinolaryngologist who referred the wife to a gastroenterologist due to her swallowing difficulties. Both physicians recommended the patients should seek help in an emergency department if their condition should deteriorate further. On day 7 the patients visited the emergency department of the medical university after a general worsening of symptoms and increasing dyspnoea in the wife. Since non of blood chemistry nor hemogram, or the performed chest X-ray revealed any pathological findings, a gastroscopy only revealed a type c gastritis and the dyspnoea resolved itself after a short time the patients were discharged the same night. On the following day (day 8) both patients presented again due to a further aggravation of symptoms. A consulting neuroophtalmologist diagnosed a paresis of the nervus abducens and voiced a suspicion for a link between the two patients. He contacted the attending infectiologist at the outpatient department. Due to the abducens paresis of the husband and the dilated pupils of both patients a suspicion for botulism developed. Both patients were referred to the outpatient department of the department of infectious diseases. Here the diagnosis “botulism” was ascertained by the attending physician: both patients presented with ocular symptoms combined with anticholinergic symptoms-typical signs of botulinum toxin type B intoxication: paresis of the nervus abducens with subsequent diplopia (♂), difficulties in accommodation resulting in blurred vision (♀), dysphagia, xerostomia, mild constipation after initial diarrhea, and urinary retention. While the symptoms first on the list are quite common in botulism, the urinary retention is a rather seldom reported aspect of the illness [1, 6–8]. To further confirm the clinical diagnosis, serum samples were sent to the reference laboratory for botulism at the Austrian Agency for Health and Food Safety (AGES) in Graz. The collection of stool and urine samples was not possible due to the urinary and stool retention of the patients.

Since the initial symptoms presented seven to ten days prior to admission, the application of botulinum antitoxin was not a therapeutic option. Botulinum antitoxin may only be used within the first 36 hours after onset of the initial symptoms. Beyond this point the toxin will already have docked irreversible to the neuromuscular endplates and removal of toxin from the bloodstream will show no effect. Although both patients presented respiratory stable, due to the nature of the intoxication often leading to a descending flaccid paralysis with need for ventilator support a conservative approach was chosen and the patients admitted to the infectious diseases ward. A neurologist was consulted who described “truly peripheral anticholinergic symptoms, combined with dysfunction of the cranial nerves and an autonomic dysfunction, without any impact on the sensible part of the neural system or any evidence for a central anticholinergic syndrome,” further reinforcing the diagnosis. A second neurologic consult on the 12th day showed a similar picture, without any improvement of the neurofunction.

In the first days on the ward both patients additionally developed epistaxis, while only the husband developed a sore throat. Since both patients had not defecated since the first signs of the intoxication (last time on the 3rd day for the male patient respectively on the 4th day for the female patient), both received treatment with lactulose syrup and in the case of the husband additional saline solution clyster to prevent paralytic ileus. This treatment was effective in both patients on the 11th respectively 12th day and had to be repeated on a regular basis of about every four days during the whole admittance from day 8 to day 22. However, the situation was alleviated after both patients received 4.5 mg metoclopramid thrice daily from the 20th day on.

After being discharged from hospital the patients underwent regular check-ups to ensure the continued benign course of the illness. Roughly four months after the initial onset of symptoms a neurological and neuroophtalmological reexamination showed a complete recovery from the illness. About one week before this check-up the wife reported a returning ability to sweat. 

**Figure 1:**
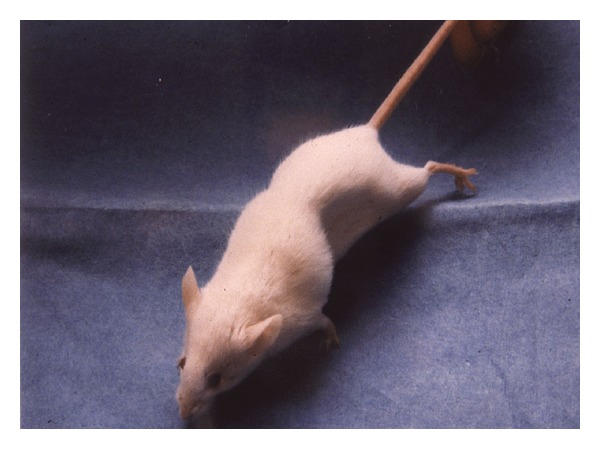
Mouse with classical “wasp waist” sign after intoxication with botulinum toxin (picture by courtesy of Professor F. Allerberger, AGES Vienna).

The reference laboratory performed a bioassay to detect botulinum toxin in the patients' sera. In this assay for each patient four mice were injected intraperitoneally with patient serum. In reaction to the toxin the mice developed a paralysis of the diaphragm, leading to a “wasp waist” (Figure 1). Two of the injected mice received an additional dose of a polyvalent botulinum antitoxin. The mice injected with the husband's serum died after 48 hours, and the mice injected with the wife's serum died after 72 hours, whereas those injected with the serum plus antitoxin did not show any symptoms, thus proving the presence of botulinum toxin in the patients sera. 

Portions of the samples were sent to the Health Protection Agency, Colindale/UK, where the toxin was confirmed as *“Clostridium botulinum *neurotoxin type B,” which is said to be mainly found in cooked pork products [8]. Preserved vegetables have been the major source of botulism in the USA from 1950 to 1996. In the years from 1990 to 1996 only 12.5% of all cases in the USA were attributed to intoxication with type B toxin, most often type A (44.6%) and E (35.7%) were the causative type [9]. The maximum reported time from intake to manifestation has been ten days [10]. Symptoms usually arise between 18 to 36 hours after ingestion. No source could be identified in the presented cases. 

During the first eight days both patients were sent to several specialists by the general practitioner as well as the emergency department before the first suspicion for botulism was voiced by a neuroophtalmologist on day 8 after exposure and the patients were referred to our department where the diagnosis “botulism” was ascertained. This case stresses out the importance of thorough anamnesis and the ability to see linked symptoms in multiple individuals. Physicians should always ask for a person with similar symptoms in the patients' vicinity if a patient presents with an unusual or unclear condition and contact a specialist for infectious diseases if this is the case.

Due to the high quality of industrial food production botulism is a disease occurring very rarely in Austria; however, especially rarely occurring cases can be the most challenging for the attending physician. In the light of the described events we think it is crucial to advert to the complex symptoms a nonsevere intoxication with botulinum toxinum can produce. Although intoxication through homemade products, often imported by visiting relatives from less developed countries, are the most likely source for botulism, the patients did stress out the fact that they had not consumed any homemade products at the time during which the intoxication must have taken place [5, 11]. Thus, in the presented case, a definitive source for the intoxication could not be found.

## Figures and Tables

**Table 1:** Cases of botulism in Austria since 1990 [3, 4].

Year	Illness	Death	Region
1996	4	0	Styria
2001	1	0	Carynthia
2004	2	0	Styria
2005	3	0	Salzburg [2], Voralberg [1]
2006	1	0	Upper Austria
2011	4	0	Carynthia [1], Vienna [2], Lower Austria [1]
